# Low expression of NCALD is associated with chemotherapy resistance and poor prognosis in epithelial ovarian cancer

**DOI:** 10.1186/s13048-020-00635-6

**Published:** 2020-03-30

**Authors:** Li-yuan Feng, Li Li

**Affiliations:** Department of Gynecologic oncology, Guangxi Medical University Cancer Hospital, 71 Hedi Road, Nanning, Guangxi 530021 P.R. China

**Keywords:** Ovarian cancer, Chemoresistance, NCALD, Prognosis

## Abstract

**Background:**

Low expression of NCALD(neurocalcin delta) in peripheral blood of ovarian cancer patients predicts poor prognosis. However, the molecular mechanism of NCALD in ovarian cancer and its relationship with chemotherapy outcomes is unclear. The aim of this study was to investigate the potential signaling pathways of NCALD and to evaluate its ability to predict chemotherapy outcomes and prognosis.

**Methods:**

High-throughput RNA sequencing data were downloaded from TCGA. GSEA explored the potential signaling pathways of NCALD. The expression of NCALD in chemotherapy sensitive and chemotherapy resistant ovarian cancer patients was detected by TCGA data and clinical samples. ROC analysis confirmed the ability of NCALD to predict chemotherapy outcomes. The association between NCALD expression and prognosis in ovarian cancer patients was assessed using Kaplan-Meier plotter.

**Results:**

In patients with NCALD overexpression, genes expression related to ERK1 / 2 signaling pathway, NF-kappaB signaling pathway, TGF-β signaling pathway and immune response pathway was increased, especially ERK1 / 2 signaling pathway. The expression of NCALD in chemoresistant patients was significantly lower than chemosensitive patients. In TCGA data and immunohistochemical samples, the AUC of NCALD expression predicting chemotherapy outcome was 0.59 and 0.64, respectively. In clinical samples, low expression of NCALD was associated with poor OS and PFS.

**Conclusions:**

NCALD may activate the ERK1 / 2 signaling pathway in ovarian cancer. As a new biomarker of chemotherapy sensitivity, NCALD was significantly down-regulated in chemotherapy resistance ovarian cancer patients. Low expression of NCALD in ovarian cancer is associated with poor OS and PFS. In the future, further research will be needed on the potential mechanism and clinical application value of NCALD in ovarian cancer.

## Background

Ovarian cancer is the most lethal gynecological malignancy. More than 75% of ovarian cancer patients are stage III or IV at the time of initial diagnosis, with a 5-year survival rate was less than 30% [1, 2]. The standard treatment plan for the initial diagnosis of ovarian cancer is optimal debulking combined with platinum-based chemotherapy [3]. Recent advances in radical surgery and chemotherapy have improved the treatment outcomes for ovarian cancer. However, chemotherapy resistance in the later stage of treatment results in high recurrence, metastasis and mortality [4, 5]. Current prognostic indicators including age, stage, and histological type are insufficient to predict the efficacy of conventional chemotherapy [6, 7]. In order to improve the prognosis of ovarian cancer patients, new biomarkers which predict chemotherapy resistance and treatment strategies to overcome resistance are needed.

NCALD(neurocalcin delta) is a member of the neuronal calcium sensor family, which is involved in calcium signaling pathway and G-protein-coupled receptor signaling pathway. It is abundant in normal brain, testis, ovary and small intestine [8, 9]. NCALD consists of four EF hand motifs, but only three can bind to calcium, which alters the conformation of the NCALD protein [10]. Studies have found that NCALD expression is associated with the prognosis of acute myeloid leukemia and non-small cell lung cancer, indicating its clinical potential as a prognosis biomarker [11, 12]. In ovarian cancer, Isaksson’s study showed that low expression of NCALD predicts poor prognosis [13]. Since Isaksson’s NACLD data are from peripheral blood. It would be important to look for the association of NACLD expression with PFS and OS to strengthen the findings. In addition, the molecular mechanism of NCALD in ovarian cancer and its relationship with chemotherapy sensitivity has not been reported. The aim of our study was to investigate the potential signaling pathways of NCALD and to evaluate its ability to predict chemotherapy outcomes and prognosis.

## Materials and methods

### TCGA data

NCALD mRNA expression (data_mRNA_median_Zscores) and clinical data of ovarian cancer in TCGA database downloaded from cbioportal (http://www.cbioportal.org/). Ovarian cancer patients without chemotherapy outcomes were excluded. Ovarian cancer patients with gender, age, grade, FIGO stage, survival outcome, chemotherapy outcome and NCALD expression were included.

### GSEA explored the potential signal pathway of NCALD in ovarian cancer

Ovarian cancer samples in TCGA were divided into low expression group and high expression group according to the NCALD expression mean. Gene enrichment analysis was performed using GSEA software (https://www.broadinstitute.org/gsea/). Number of permutation = 2500, gene sets with *P* values < 0.005 and FDR q values < 0.20 are considered to be significantly enriched gene sets.

### Patient samples

From March 1997 to April 2013, 178 epithelial ovarian cancer patients were collected from the Guangxi Medical University Cancer Hospital. One hundred seventy-eight paraffin tissues were used for immunohistochemistry, and 61 frozen tissues were used for QRT-PCR. The inclusion and exclusion criteria are as follows. Inclusion criteria: (1) patients with chemotherapy outcomes; (2) patients with postoperative pathology; (3) patients with survival outcomes; and (4) patients with complete clinical data (including gender, age, grade and FIGO stage). Exclusion criteria: (1) patients without definite postoperative pathology; (2) patients without complete data (including chemotherapy outcomes, survival outcomes and clinical data). All Samples received the patient’s informed consent and the approval of the Ethics Committee of the Guangxi Medical University Cancer Hospital.

Chemoresistance was defined as the patients who do not achieve a complete response after initial treatment, or with relapse within 6 months after complete response. Chemosensitivity was defined as patients with recurrence more than 6 months after complete response. Overall survival (OS) was defined as the time from diagnosis to death due to ovarian cancer. Progression-free survival (PFS) was defined as the time from initial treatment to tumor progression.

### RNA extraction and QRT-PCR

RNA extraction and reverse transcription were performed according to the Genomic RNA Purification Kit (Thermo Scientific, Cat. No. K0731) and Revert Aid First Strand cDNA Synthesis Kit (Thermo Scientific, Cat. No. K1622) instructions. One Step TB Green® PrimeScriptTM RT-PCR Kit (Takara, Cat. No. RR066B) was used for real-time PCR on an ABI step-one plus PCR machine.

### Tissue microarray and immunohistochemistry

The pathological types were confirmed by HE stain. Each tumor has 2–3 repeated tissue spots. The diameter is 1 mm. Immunohistochemistry was performed according to the ready-to-use immunohistochemical ultrasensitive UltraSensitiveTM SP test kit (maixin, Cat. No. KIT-9710) instructions. NCALD concentration is 1:400 (Abcam, Cat. No.ab155161). Two pathologists read the pathological sections independently. The score criteria are as follows: Positive cell ratios of < 1, 1–25%, 25–50%, 50–75% and 75–100% were assigned 0, 1, 2, 3, 4 points, respectively. Stain intensity of no coloring, light yellow, yellow, brown were assigned 0, 1, 2, 3 points, respectively. The product of positive cell ratio and stain intensity is stain index. Stain index≤6 points was classified as low expression, while > 6 points was classified as high expression [14].

### Statistical analysis

SPSS17.0 was used for analysis. T test (measurement data) and chi-square test (categorical data) were used for comparison between the two groups. ROC analysis confirmed the ability of NCALD to predict chemotherapy outcomes. The association between NCALD expression and prognosis was assessed using Kaplan-Meier plotter.

The association between NCALD expression and clinicopathological parameters was assessed with Spearman test. *P* values were two-sided, and *P* < 0.05 was considered statistically significant.

## Results

### Characteristics of patients

Data from 491 ovarian cancer patients were downloaded from the TCGA, and 204 patients without chemotherapy outcomes were excluded. 287 ovarian cancer patients with gender, age, grade, FIGO stage, survival outcome, chemotherapy outcome, and NCALD mRNA expression were obtained. The clinical characteristics are shown in Table 1. Among the 178 clinical samples, 34 had FIGO stage I or II and 134 patients had FIGO stage III or IV. There were 141 patients with grade 3 and 22 patients with grade 1–2. There were 129 patients with serous type and 49 patients with other histology types.

**Table 1:** Clinical characteristics of patients

	TCGA(*N* = 287)				IHC(*N* = 178)			
Clinical characteristics	All (287)	chemoresistant (90)	chemosensitive (197)	*P*	All (178)	chemoresistant (71)	chemosensitive (107)	*P*
FIGO stage								
I-II	14 (4.88%)	1 (1.11%)	13 (6.60%)	0.088	38 (22.09%)	8 (11.76%)	30 (28.85%)	0.008^*^
III-IV	273 (95.12%)	89 (98.89%)	184 (93.40%)		134 (77.91%)	60 (88.24%)	74 (71.15%)	
Missing	0	0	0		6	3	3	
Grade								
3	243 (86.48%)	81 (91.01%)	162 (84.38%)	0.130	141 (86.50%)	57 (86.36%)	84 (86.60%)	0.989
2	38 (13.52%)	8 (8.99%)	30 (15.62%)		16 (9.82%)	7 (10.61%)	9 (9.28%)	
1	0	0	0		6 (3.68%)	2 (3.03%)	4 (4.12%)	
Missing	6	1	5		15	5	10	
Histology types								
Serous	287	90 (100.00%)	197 (100.00%)	–	129 (72.47%)	46 (64.79%)	83 (77.57%)	0.078
Mucinous	0	0 (00.00%)	0 (00.00%)		10 (5.62%)	6 (8.45%)	4 (3.74%)	
Others	0	0 (00.00%)	0 (00.00%)		39 (21.91%)	19 (26.76%)	20 (18.69%)	
Surgical debulking								
Optimal	56 (21.54%)	9 (10.59%)	47 (26.86%)	0.003^*^	71 (64.55%)	28 (53.85%)	43 (74.14%)	0.026^*^
Suboptimal	204 (78.46%)	76 (89.41%)	128 (73.14%)		39 (35.45%)	24 (46.15%)	15 (25.86%)	
Missing	27	5	22		68	19	49	

Note: *p* value was compared between the chemosensitive patients and the chemoresistant patients, “*” means *p* < 0.05

Optimal surgical debulking rate was 21.54% (56/260) and 64.55% (71/110) in TCGA data and clinical samples, respectively. In TCGA and clinical samples, the number of patients with chemotherapy resistance who achieved optimal surgical debulking was significantly less than patients with chemotherapy sensitivity (TCGA: 9 vs 47, clinical samples: 28 vs 43). In clinical samples, the number of chemotherapy resistant patients with stage III-IV was significantly higher than stage I-II (8 vs 30), but there was no difference in TCGA data.

### Potential signal pathways of NCALD in ovarian cancer

**Fig. 1:**
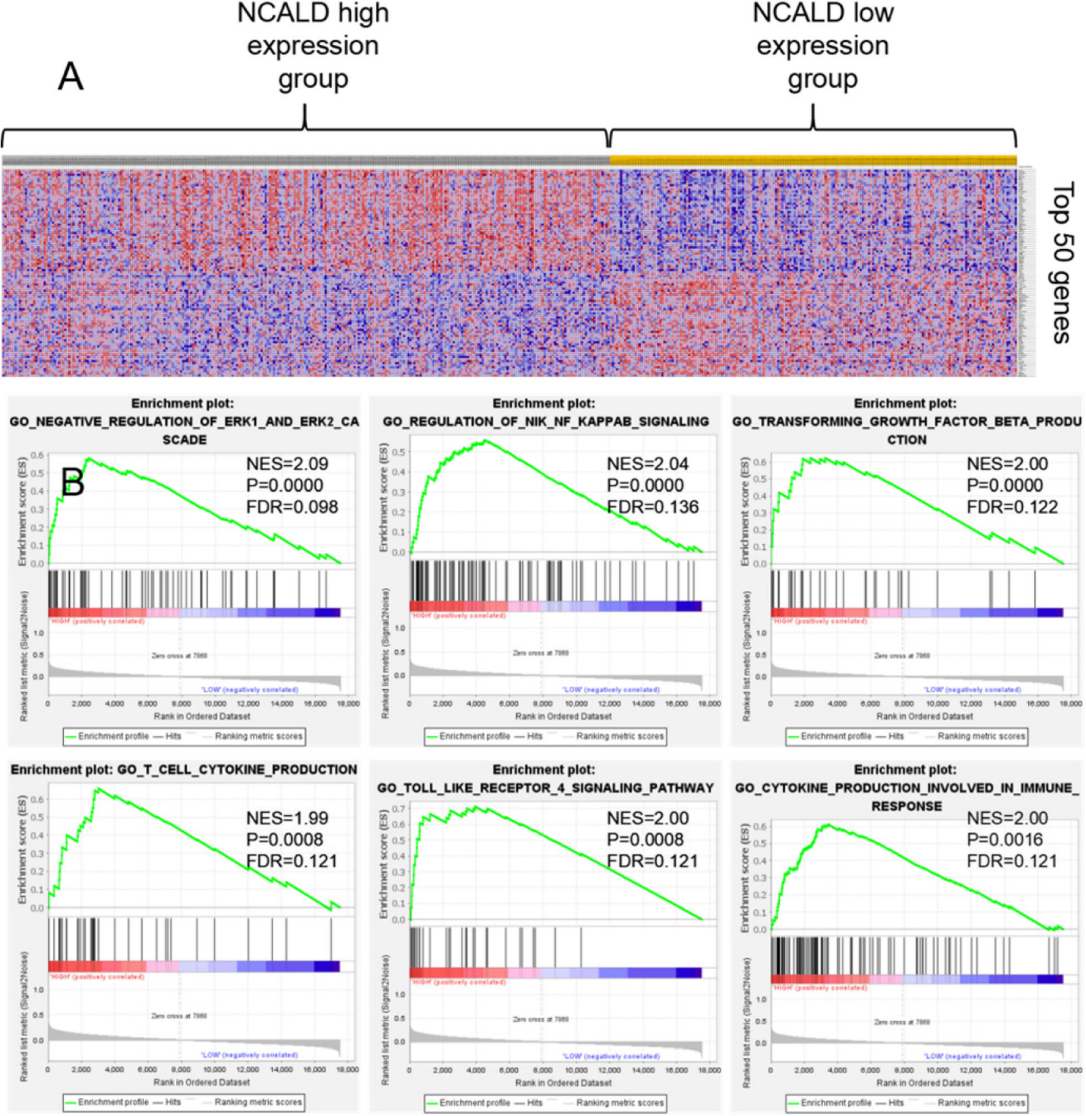
**a** Top 50 genes that are highly expressed in ovarian cancer. **b** NCALD related key signaling pathways

Four hundred ninety-one Ovarian cancer samples in TCGA were divided into low expression group and high expression group according to the NCALD expression mean. The transcriptome data between 294 patients with NCALD high-expression and 197 patients with NCALD low-expression was compared. The key cellular processes and signal pathways of NCALD in ovarian cancer were identified by gene set enrichment analysis (GSEA). There were 17,518 differentially expressed genes in the two groups, 7868 (44.90%) genes were highly expressed in the NCALD high-expression group, and 9650 (55.10%) genes were highly expressed in the NCALD low-expression group. The complete list of differentially expressed genes is shown in supplementary document 1. The heat map shows the top 50 genes that are highly expressed in the NCALD overexpression group and the low-expression group, respectively, see Fig. 1a.

Gene set enrichment analysis (GSEA) showed that the genes expression related to ERK1/2 signal pathway, NF-kappaB signal pathway, TGF-β signal pathway, and immune response pathway was increased in the NCALD overexpression group, especially ERK1 / 2 signaling pathway (NES = 2.09). See Fig. 1b. The complete list of differential gene set is shown in supplementary document 2.

### Expression of NCALD in chemosensitive and chemoresistant ovarian cancerpatients in TCGA data

**Fig. 2:**
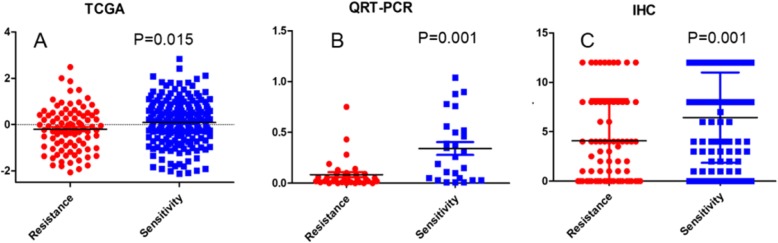
**a** Expression of NCALD in TCGA data. **b** Expression of NCALD in clinical samples (QRT-PCR). **c** Expression of NCALD in clinical samples (IHC)

The expression of NCALD in chemoresistant patients was significantly lower than that in chemosensitive patients. The mean ± standard deviation was − 0.21 ± 0.94 VS 0.09 ± 0.95, see Fig. 2a. Spearman correlation analysis showed no correlation between NCALD expression and FIGO stage, grade and residual disease. Data are not shown.

### Expression of NCALD in chemosensitive and chemoresistant ovarian cancer patients in clinical samples

Clinical samples were used to further verify the expression of NCALD in chemosensitive and chemoresistant ovarian cancer patients. QRT-PCR results showed that the expression of NCALD in chemoresistant ovarian cancer patients was significantly lower than that in chemosensitive ovarian cancer patients (0.08 ± 0.15 VS 0.34 ± 0.32), see Fig. 2b.

**Fig. 3:**
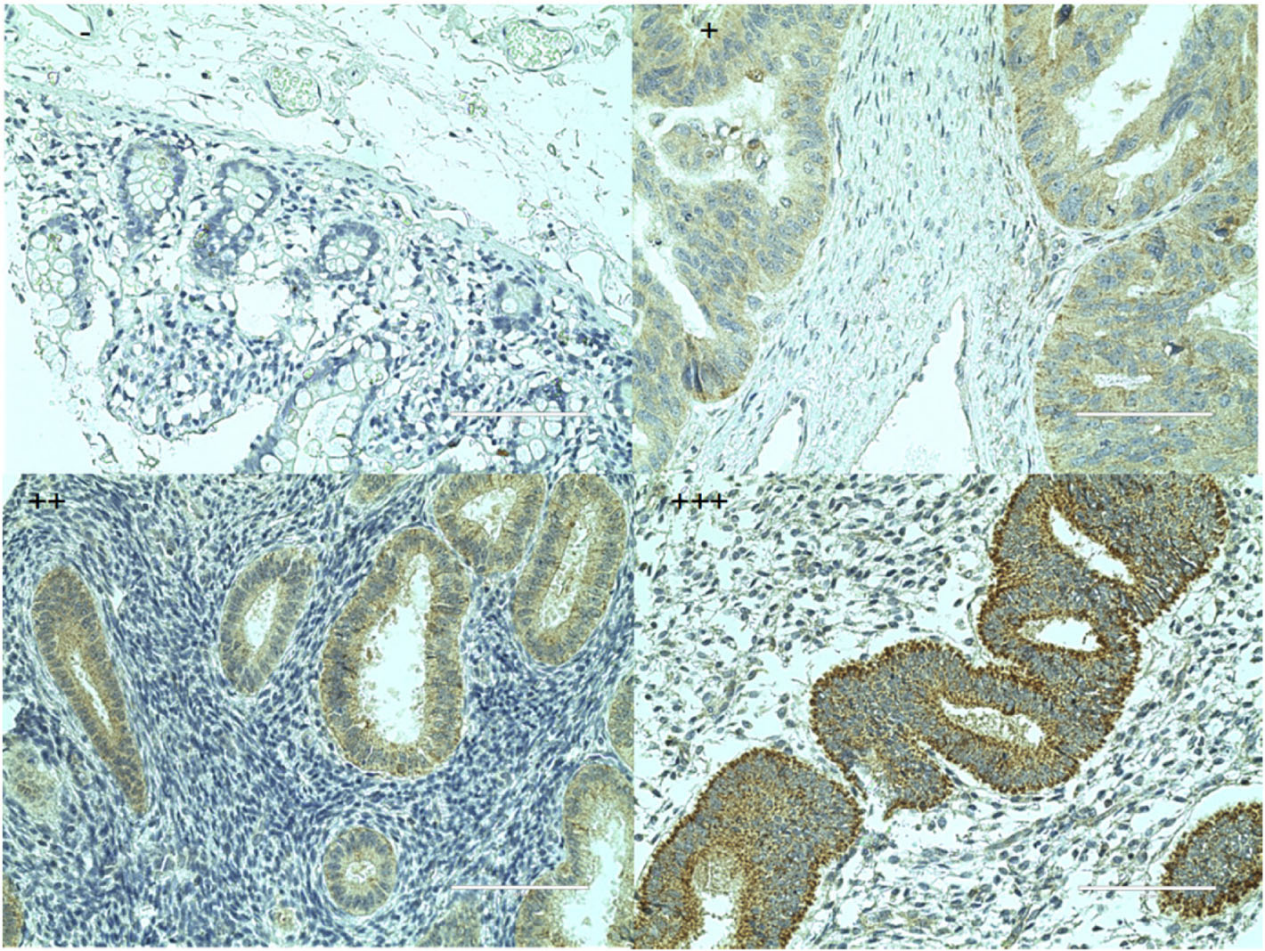
Immunohistochemical stain (400×)

Immunohistochemistry results showed that NCALD is mainly expressed in the cytoplasm, and a small amount is expressed in the cell membrane. NCALD was highly expressed in 59/107(55.14%) chemosensitive patients and 21/71(29.58%) chemoresistant patients. A chi-square test showed that the protein expression of NCALD in chemoresistant patients was significantly reduced compared with chemosensitive patients, as showed in Table 2 and Fig. 2c. Immunohistochemical stain is shown in Fig. 3.

**Table 2:** Relationship between NCALD expression and chemotherapy outcome in epithelial ovarian cancer

	TCGA				QRT-PCR				IHC					
	Patients	N	Expression	*P*	Patients	N	Expression	*P*	Patients	N	Low expression(N)	High expression(N)	Positive rate	*P*
Expression of NCALD	chemoresistant	90	−0.21 ± 0.94	0.015*	chemoresistant	35	0.08 ± 0.15	0.001*	chemoresistant	71	50	21	29.58%	0.001*
	chemosensitive	197	0.09 ± 0.95		chemosensitive	26	0.34 ± 0.32		chemosensitive	107	48	59	55.14%	

Note: continuous data are represented by means±standard deviations, while classified data are represented by values and percentages. “*” means *p* < 0.05

### Stratified analysis

In clinical samples, stratified analysis results showed that in FIGO III-IV stage patients, poorly differentiated patients and other epithelial histology types (except serous and mucinous) patients, NCALD expression in chemotherapy sensitive patients was higher than that in chemotherapy resistant patients (54.05% VS 30.00%, *P* = 0.005; 59.00% VS 33.33%, *P* = 0.002; 35.00% VS 00.00%, *P* = 0.015). There was no significant difference in the stratified analysis of the TCGA data. See supplementary document 3.

### The ability of NCALD to predict chemotherapy outcomes

**Fig. 4:**
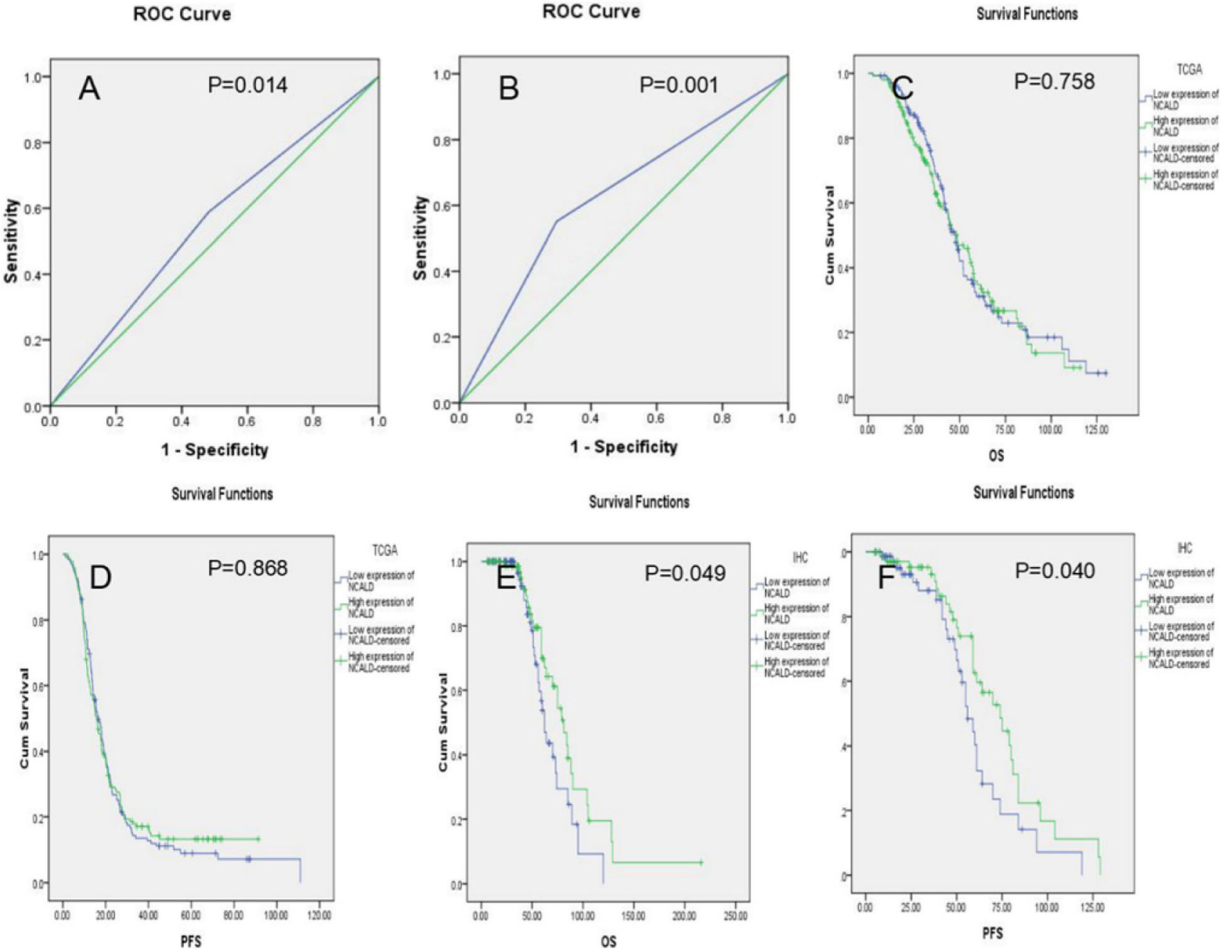
**a** ROC curve in TCGA data. **b** ROC curve in clinical samples. **c** Association of NACLD expression with OS in TCGA data. **d** Association of NACLD expression with PFS in TCGA data. **e** Association of NACLD expression with OS in clinical samples (IHC). **f** Association of NACLD expression with PFS in clinical samples (IHC)

In TCGA data and clinical samples, ROC analysis showed that the sensitivity, specificity, accuracy and AUC of NCALD expression to predict chemotherapy sensitivity were 0.70, 0.46, 0.53, 0.59(95%CI 0.52–0.66) and 0.59, 0.68, 0.61, 0.64(95%CI 0.56–0.73) respectively. The ROC curve was shown in Fig. 4a and b. The positive likelihood ratio and negative likelihood ratio was shown in Table 3.

**Table 3:** The ability of NCALD to predict chemotherapy outcomes in epithelial ovarian cancer

NCALD expression	AUC	*P*	Youden	Sensitivity	Specificity	Accuracy	Positive likelihood ratio	Negative likelihood ratio
TCGA	0.59 (95%CI 0.52–0.66)	0.014*	0.16	0.70	0.46	0.53	1.28	0.67
IHC	0.64 (95%CI 0.56–0.73)	0.001*	0.27	0.59	0.68	0.61	1.82	0.61

Note: “*” means *p* < 0.05. Youden = sensitivity + specificity-1

### Low expression of NCALD is associated with poor OS and PFS

In the TCGA data, Kaplan-meier survival analysis showed that OS and PFS were shorter in patients with low expression of NCALD, but the differences were not statistically significant (OS:48.72(95%CI 39.27–58.17) VS 47.37(95%CI 42.55–52.19); PFS:16.59(95%CI 14.25–18.93) VS 15.77(95%CI 13.16–18.38)). In clinical samples, patients with low expression of NCALD were associated with poorer OS and PFS (OS:81.00(95%CI 68.18–93.82) VS 62.00(95%CI 55.76–68.24); PFS:74.00(95%CI 58.32–89.68) VS 56.00(95%CI 49.02–62.98)). The survival curve is shown in Fig. 4c-f.

## Discussion

Chemotherapy resistance is the main clinical obstacle in the treatment of ovarian cancer [15]. There is an urgent need to explore new biomarkers that can predict chemotherapy resistance early and therapeutic strategies to reverse resistance. NCALD is a member of the neuronal calcium sensor family. It involves in neuronal diseases and nutritional metabolic diseases. Such as spinal muscular atrophy [16–18], epilepsy [19, 20], depression [21], obesity [22], diabetes [23] and celiac disease [24]. Currently, there is little research on NCALD in ovarian cancer. Our study explored the potential signaling pathways of NCALD and evaluated its ability to predict chemotherapy outcomes and prognosis.

Our GSEA results showed that the genes expression related to ERK1/2 signal pathway, NF-kappaB signaling pathway and immune response pathway were increased in the NCALD overexpression ovarian cancer patients. ERK1 (44 kDa) and ERK2 (42 kDa) are homologous subtypes of ERK family and have the same substrate specificity [25, 26]. Erk1/2 is an important component of the cascade of Ras-Raf-MEK-ERK signaling pathway, which has received extensive attention in tumor resistance and tumor therapy [27–29]. It is activated in many cisplatin treated cell lines [30–33]. In the resting state, ERK1/2 binds to MEK in the cytoplasm. After phosphorylation, p-ERK1/2 is transferred to the nucleus and regulates the activity of some transcription factors, such as: c-fos, c-Jun, Elk-1, STATs, c-myc, NF-jB. Through the abnormal activation of ERK signal, genetic alterations in Ras or RAF family members lead to rapid tumor growth and resistance to apoptosis, which resulting in chemotherapy resistance [34–36]. NF-kB (transcripton factor nuclear kappa B) is an important mediator that acts in the chronic inflammaton [37]. NF-kB was highly expressed in ovarian cancer and related to ovarian cancer progression [38, 39] . Immunotherapies attenuate the levels of nuclear factor kappa B, reduce cell dynamics and effectively target the TLR-related downstream molecules, eliciting a protective effect against chemoresistance [40].

We analyzed the relationship between NCALD expression and chemotherapy outcomes in ovarian cancer patients. First, TCGA data showed that NCALD expression significantly decreased in chemotherapy resistant patients compared to chemotherapy sensitive patients. Second, in local clinical samples, QRT-PCR and immunohistochemical results confirmed that NCALD expression was significantly reduced in chemotherapy resistant patients compared to chemotherapy sensitive patients. Third, stratified analysis showed that in FIGO III-IV stage patients, grade 3 patients and other histology types (except serous and mucinous) patients, NCALD expression in chemotherapy sensitive patients was higher than that in chemotherapy resistant patients. In TCGA data and immunohistochemical samples, the AUC of NCALD expression predicting chemotherapy outcome was 0.59 (95% CI 0.52–0.66) and 0.64 (95% CI 0.56–0.73), respectively. NCALD expression has certain diagnostic value for chemosensitivity.

In TCGA data, NCALD expression was not significantly correlated with OS and PFS. But in local clinical samples, patients with low expression of NCALD were associated with poorer OS and PFS, confirming Isaksson’s findings. We found that in TCGA and clinical samples, the number of patients with chemotherapy resistance who achieved optimal surgical debulking was significantly less than patients with chemotherapy sensitivity. Residual lesions are associated with the risk of chemoresistance. Rauh-Hain’s study showed that residual tumor mass > 1 cm was a risk factor associated with the risk of platinum resistance recurrence [41]. Luo retrospectively analyzed 341 patients with FIGO III-IV stage epithelial ovarian cancer patients. Postoperative residual tumor mass > 1 cm was also found to be a risk factor for platinum resistance recurrence (OR = 2.92;95%CI 1.78–4.77,*P* = 0.000) [42]. It can be seen that our results are consistent with research by Rauh-Hain and Luo.

There are several shortcomings in this study. First, bioinformatics results showed that the genes expression related to ERK1/2 signal pathway was increased in the NCALD overexpression ovarian cancer patients. This results need to be verified by confirmatory studies. Second, the ROC curves, while having some predictive value, are not very impressive. Predicting chemotherapy resistance is difficult, especially for single biomarker. It will be necessary to further improve the predictive efficacy by combining with other biomarker.

## Conclusions

In summary, our study demonstrates that NCALD may activate the ERK1 / 2 signaling pathway in ovarian cancer. As a new biomarker of chemotherapy sensitivity, NCALD was significantly down-regulated in chemotherapy resistance ovarian cancer patients. Low expression of NCALD in ovarian cancer is associated with poor OS and PFS. In the future, further research will be needed on the potential mechanism and clinical application value of NCALD in ovarian cancer.

## Supplementary information

**Additional file 1: Supplementary document 1.** The complete list of differentially expressed genes. **Supplementary document 2.** The complete list of differential gene set. **Supplementary document 3** Stratified analysis.

## Data Availability

Not applicable.

## Abbreviations

AUC: Area Under Curve
FIGO: The international federation of gynecology and obstetrics
GSEA: Gene Set Enrichment Analysis
NCALD: Neurocalcin delta
OS: Overall survival
PFS: Progression-free survival
ROC: Receiver Operating Characteristic
TCGA: The Cancer Genome Atlas
